# Conservation Weighting Functions Enable Covariance Analyses to Detect Functionally Important Amino Acids

**DOI:** 10.1371/journal.pone.0107723

**Published:** 2014-11-07

**Authors:** Lucy J. Colwell, Michael P. Brenner, Andrew W. Murray

**Affiliations:** 1 University Chemical Laboratory, Cambridge University, Cambridge, United Kingdom; 2 School of Engineering and Applied Sciences, Harvard University, Cambridge, Massachusetts, United States of America; 3 FAS Center for Systems Biology, Harvard University, Cambridge, Massachusetts, United States of America; CRG, Spain

## Abstract

The explosive growth in the number of protein sequences gives rise to the possibility of using the natural variation in sequences of homologous proteins to find residues that control different protein phenotypes. Because in many cases different phenotypes are each controlled by a group of residues, the mutations that separate one version of a phenotype from another will be correlated. Here we incorporate biological knowledge about protein phenotypes and their variability in the sequence alignment of interest into algorithms that detect correlated mutations, improving their ability to detect the residues that control those phenotypes. We demonstrate the power of this approach using simulations and recent experimental data. Applying these principles to the protein families encoded by *Dscam* and *Protocadherin* allows us to make testable predictions about the residues that dictate the specificity of molecular interactions.

## Introduction

Determining which residues of a protein control its biological functions is a classical and unsolved problem in molecular biology. For example the biochemistry of allosteric enzymes has long been studied, but it is not in general known which residues produce the allosteric response, even for proteins that have been exceedingly well studied such as hemoglobin [1]–[3]. The growth in the number of available sequences has given rise to the intriguing possibility of using the phenotypic diversity contained in multiple sequence alignments (MSAs) to address this question [4], [5]. Given both a sequence alignment containing a large number of homologous proteins, and a phenotype of interest, can an algorithm be developed to identify those residues that control this phenotype? By phenotype we mean the functional properties of a protein, such as melting temperature, interaction partners, or substrate specificity. Since protein phenotypes such as these are often controlled by a collection of residues, it is unlikely that patterns of individual mutations contain enough information to identify residues controlling the functional variation between different members of the same family [1], [6]–[8].

A pair of algorithms, featured in a number of recent papers, have provided compelling experimental evidence that detection of correlated pairs of residues can identify groups of residues that control different protein phenotypes [7]–[14]. Using statistical coupling analysis (SCA) Halabi *e*t al. identify groups of residues that control the structural stability and enzyme activity of the serine proteases [8]. SCA analysis was recently used to identify residues involved in the control of allosteric regulation both within and between protein domains [10], [12] and residues important for both function and adaptation [11]. In addition, using mutual information (MI) Skerker *e*t al. identify specificity-determining residues in bacterial signal transduction proteins [7], [9], [13], [14]. These sets of studies carry out extensive experiments to validate their predictions, which are obtained using two different algorithms to detect correlated residue pairs. To test the importance of the choice of algorithm, we repeated the analyses in [7], [8] with the algorithms swapped, namely using mutual information to analyze the serine proteases, and SCA to analysis the signal transduction proteins. We find that the algorithms are not interchangeable, implying that the ability to detect correlated mutations in these studies depends on the details of each algorithm. For such analyses to be applicable to other biological datasets, we need to understand which properties of the algorithm determine its effectiveness, and design a more general algorithm based on these principles.

Both algorithms are based on the idea of detecting correlated mutations between residues in sequence alignments. This is a sound approach, because if a phenotype is controlled by a set of residues, members of the set must mutate to change the phenotype, and therefore, these residues can be detected by looking for groups of sequence positions whose mutations are correlated. Many statistical measures have been suggested that quantify the degree of correlation between sequence positions in a multiple sequence alignment, and different authors have suggested weighting these raw correlation scores in different ways [7], [13], [15]–[22]. In particular, mutual information and SCA use different metrics for measuring the raw correlation score, and in addition these metrics are differently weighted.

This manuscript is organized as follows. We first identify the critical difference that keeps SCA and mutual information from being interchangeable algorithms, which turns out to be the different weights applied to the raw correlation scores. To create an algorithm that works more generally we propose using biological information about the expected conservation level of the phenotype in question to design context specific weighting functions. This approach performs well on both original datasets, so we turn to testing it in more general situations. We first demonstrate that the algorithm performs well on artificial sequences generated through simulations of a simple model of molecular evolution, in which the conservation level of the phenotype is systematically varied. We then demonstrate that it performs well on a biological example (Dscam domains) in which the phenotype controlling residues have been identified through experiments. Finally, we make testable predictions by applying our algorithm to Cadherins and Protocadherins for which the phenotype-controlling residues have not yet been probed experimentally.

## Results

We start by focusing on two experimental studies: Skerker *e*t al. use mutual information (MI) to identify residues that control interaction specificity between cognate histidine kinases (HKs) and response regulators (RRs) [7], while Halabi *e*t al. use statistical coupling analysis (SCA) to identify groups of residues that control the enzyme activity and structural stability of the serine proteases [8]. In both cases extensive experimental work showed that the predicted residues indeed control the phenotype of interest.

**Figure 1:**
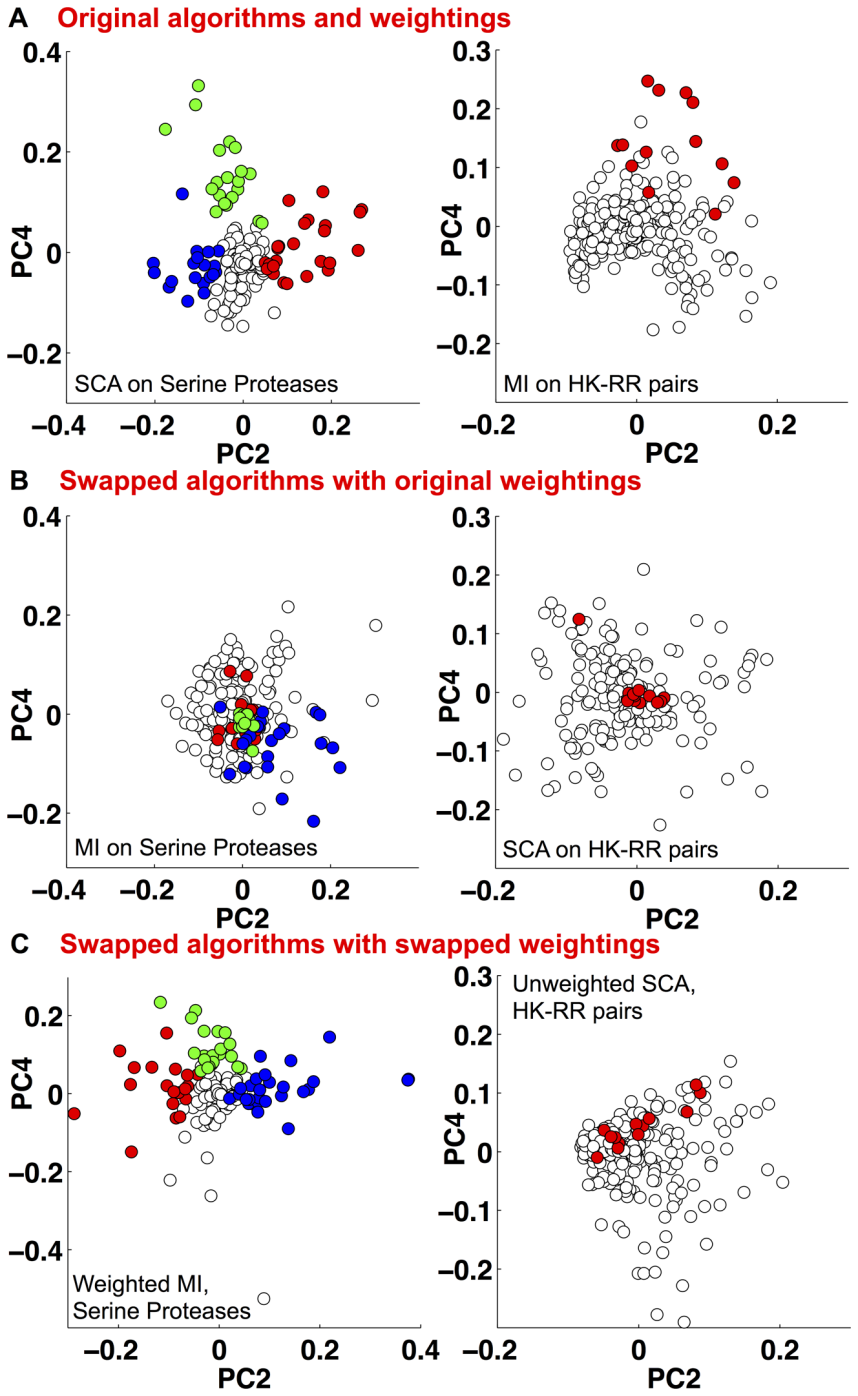
A) PCA of (left) the correlation matrix produced by SCA v3.0 applied to the serine protease alignment and (right) the correlation matrix produced by MI applied to the histidine kinase - response regulator (HK-RR) alignment (see methods). These plots largely recover the experimentally verified residues (red, green and blue) that control the different phenotypes identified in [8] and [7] respectively. B) (Left) Applying MI to the serine protease alignment does not recover the functionally relevant residues, colored as (A), they mostly cluster around the origin. (Right) Applying SCA to the HK-RR sequence alignment also does not recover the relevant residues. C) (Left) Applying MI combined with the SCA weighting function to the serine protease alignment recovers the relevant residues, compare with A. (Right) Application of unweighted-SCA to the HK-RR alignment improves performance at detecting the relevant residues.

We examined whether the same predictions would be made if the algorithms used by these two groups were swapped. We first ran the original algorithms on the original alignments, that is SCA for the serine proteases, and MI for the HK-RRs, and used principal component analysis (PCA) to generate the plots shown in Fig. 1A. In [8] it is argued that the principal components define three groups of residues, distinguished by the coefficients of the second and fourth principal component, as shown on the left of Fig. 1A (colored according to [8]). Strikingly, one of these groups contains the catalytic triad and associated residues. The right panel of Fig. 1A shows our PCA analysis of the correlation matrix from [7], verifying that the specificity determining residues found in [7] and colored in red are grouped together, away from the origin. Note that PCA was not used to identify residue pairs in [7].

Fig. 1B shows the result of switching the algorithms for these two alignments. On the left we apply MI to the serine protease alignment from [8]; the residues are colored as before. Many of the colored residues shown to be functionally important in [8] lie close to the origin of this plot; other PC combinations also fail to recover the separation between the three functional sectors (Fig. S1 in file S1). On the right of Fig. 1B we apply SCA to the HK-RR alignment from [7]; the specificity determining residues, validated in [7], are highlighted in red. This figure, together with Fig. S2 in file S1, shows that SCA is unable to discriminate these residues from others. Thus, each algorithm is only able to correctly identify the important residues from one alignment.

There are two major differences between these two algorithms: the change in statistical method for detecting correlation and the weighting function used in SCA (see methods, Fig. S3A in file S1). We write the covariance matrix as 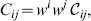
(1)where 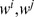 is a weighting function and 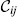 a metric for the raw correlation between residues 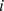 and 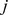. The SCA algorithm uses a weighting function 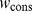 that upweights conserved residues (see methods) and correlation measure 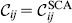 while the MI algorithm uses a weighting function 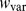 that upweights variable residues and 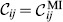.

A critical test is whether applying the SCA weighting function to MI, creating a hybrid ‘weighted-MI’ algorithm, can uncover the sectors that were experimentally validated in [8]. Figure 1C shows PCA of the correlation matrix generated by applying this new algorithm to the serine protease alignment from [8]. Comparing the left panel of Fig. 1C with that of Fig. 1A, we see both algorithms are able to identify the groups of phenotype-controlling residues verified in [8]. Similarly, the right panels of Fig. 1C and Fig. 1A reveal that the hybrid ‘unweighted-SCA’ better identifies the residues shown to control specificity in the HK-RR alignment from [7], although unweighted SCA clearly performs worse than MI on this alignment. In Fig. S3 in file S1 we further demonstrate that changing the weighting function changes the set of residues that are identified. Thus to a great extent the choice of weighting function, rather than the statistical method used, determines identification of the phenotype-controlling residues.

Our analysis finds that use of a weighting function specific to the phenotype and sequence set of interest is crucial to successful identification of phenotype-controlling residues. While perhaps surprising, this observation has a natural theoretical basis. The challenge is to identify residue pairs that are correlated to maintain a phenotype such as binding specificity or tertiary structure [4], [7], [8]. To first order, residues that control a phenotype will change when the phenotype changes. Hence, these residues will most likely have a similar conservation level to the phenotype itself in the sequence alignment. By weighting the pairwise correlation scores by a function of conservation that peaks at this level, our approach allows biological information to be incorporated into a correlated mutation analysis. This weighting function should thus be tuned to the phenotype and set of sequences of interest.

**Figure 2:**
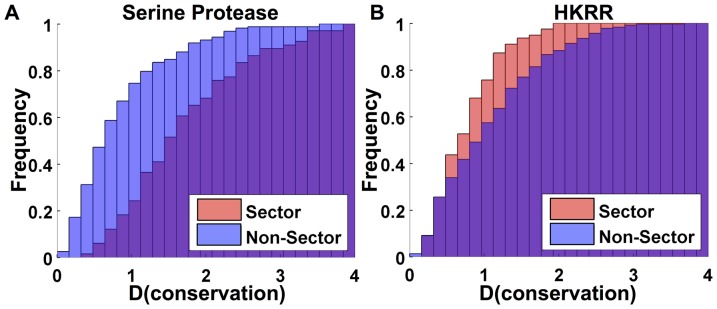
Amino acid conservation measured using the function *D* (see methods). A) Residues identified by SCA analysis of the serine protease alignment (red, labelled sector) are more conserved than the remaining residues (blue, labelled non-sector, overlap is purple). B) Residues identified by MI analysis of the HK-RR family alignment (red, labelled sector) are less conserved than the remaining residues (blue, labelled non-sector).

Indeed, a direct examination of the conservation level, defined by the function 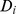 (Eqn. (4)), of phenotype determining residues shows a substantial difference between the two examples. Fig. 2A plots the conservation level of residues in the serine protease alignment; on average those residues identified by SCA (red) are more conserved than residues not included in any sector (blue, overlap of red and blue is purple). In contrast, Fig. 2B shows that residues that determine the specificity of HK-RR interaction, identified by MI, are on average more variable than other residues.

Importantly, these conclusions are as expected based on our prior knowledge of the biology of these two protein families. Because the serine protease alignment contains members of a well-conserved family of enzymes, we expect the phenotype determining residues to be more conserved, on average, than other residues. The weighting function 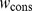 used in SCA highlights these residues, identifying three groups in the serine proteases [8]: (i) the catalytic triad, well conserved amongst the proteases but absent from the haptoglobins, making up 5% of the alignment; (ii) the catalytic site support network, which discriminates between different enzyme types (trypsins, chymotrypsins, etc.) and requires substantial coordination to keep the proteins catalytically active, and (iii) the network suggested to form the essential core needed for protein folding and stability, which is likely to require conservation to allow the protein to achieve a unique, folded structure. In contrast, the phenotype of interaction specificity among the histidine kinase response regulator pairs is highly variable, and 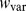 used by MI does not highlight conserved residues. Here, the protein interaction interface lies at the surface of two well-folded, globular proteins; its only role is to enable the proteins to bind in the correct orientation for phosphate transfer. Since different pathways in the same cell must avoid cross-talk, there is selection for the different specificities to be well-dispersed in sequence space [9].

The fact that biological knowledge about sequence alignments is often available suggests a general method for using this information to design weighting functions. Namely, since we want to focus our analysis on the residues whose conservation level matches that of the phenotype in the alignment of interest, we must choose the weighting function to upweight the scores of these residues. If the phenotype determining residues are expected to be highly variable (conserved), the weighting function should focus on residues that are correlated and highly variable (conserved). To implement this, we propose that the weighting function (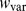) used for the response regulator pairs is applied to cases where highly variable phenotypes are expected, and similarly, the weighting function (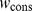) used for the serine protease is applied for more conserved phenotypes.

We now test this algorithm in several different situations, including simulations of artificial sequences and sequence alignments of protein domains for which the phenotype determining residues are known.

### Tests with Simulation

**Figure 3:**
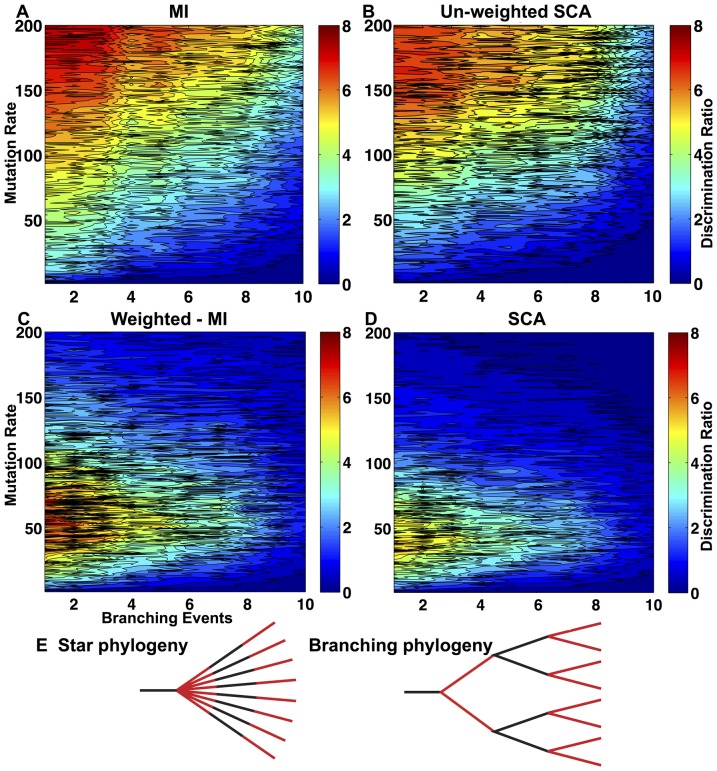
Comparison of algorithm performance on simulations of molecular evolution where 10 residue pairs are correlated while 80 residues mutate independently. (A) The MI algorithm, consisting of weighting function 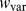 and 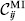; (B) The unweighted SCA algorithm, consisting of weighting function 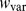 and 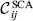 (we call this algorithm “unweighted” since 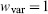) (C) The weighted MI algorithm, consisting of weighting function 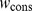 and 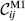; and (D) The SCA algorithm, consisting of weighting function 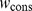 and 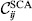. Each figure shows the discrimination ratio (the ratio of the lowest correlated score to the highest uncorrelated score) for each MSA analyzed, as a function of the mutation rate and phylogeny. Note that the major difference between these algorithms is caused by the weighting function, *not* by the functional form of 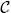. (E) The phylogeny was varied by changing the number of branching events that occurred. The star phylogeny has one branching event, while the branching phylogeny displayed here has three.

We generated a set of test sequence alignments using a simple molecular model of evolution. Most amino acids evolve independently through a Markov model whose mutation matrix is derived from BLOSUM90 [23], while we explicitly correlate the mutation of a small set of residue pairs. We vary two alignment properties: the average mutation rate and the phylogenetic tree according to which the sequences are generated. This is parameterized by the number of duplication events that occur, ranging from 1 for a star phylogeny to 10 for a maximally branched tree. To quantify how well each algorithm discriminates between correlated and uncorrelated residues, we define a metric by dividing the lowest correlation score assigned to a correlated pair by the highest score assigned to a pair that mutates independently. In Fig. 3 low scores indicate poor discrimination (dark blue), while high scores indicate excellent discrimination (red).

As expected, the performance of algorithms using 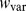 increases monotonically with mutation rate and decreases as the phylogeny becomes more complicated. In contrast, the performance of algorithms using 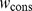 peaks when the number of duplication events is small, but the mutation rate is intermediate. This establishes (Figs. 3C and 3D) that the choice of weighting function, rather than the formula used to measure correlation (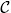) dominates the algorithm performance. All algorithms perform worse as the level of branching in the phylogeny rises because mutations in the uncoupled residues that occurred on the same branch of a phylogeny produce spurious correlations, and the strength of these correlations increases with the depth of the branch.

To test the impact of conservation on detecting coupling, we set the mutation rate of the coupled residues to be either higher or lower (Figs. S4A,B in file S1) than that of residues that are not correlated. We find that algorithms using 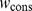 detect correlated pairs more reliably when they are more conserved than uncorrelated pairs.

### Tests with Biological Data

We now apply the method to a number of biological datasets. We start with examples in which the phenotype determining residues have been experimentally determined, and demonstrate that the algorithm is able to recover these results. The *Dscam* gene gives rise to thousands of different protein isoforms whose ability to homodimerize specifically guides neuronal wiring [24]. There are 12, 48, and 33 alternatives at Ig domains 2, 3, and 7 respectively that can be included in any individual isoform. For both the Ig2 and Ig3 domains a group of residues has been experimentally shown to determine homodimerization specificity, while for the Ig7 domain specificity determining residues have been inferred from the 3d structure [24]–[26]. We applied both weighting functions to alignments of these three variable domains [27]. On the basis of biological knowledge about the function of the proteins, we expect that there is likely strong selection for diversity at the residues that determine interaction specificity, and hence we would expect a weighting function that preferentially detects variable residues to best identify the specificity determining residues.

**Figure 4:**
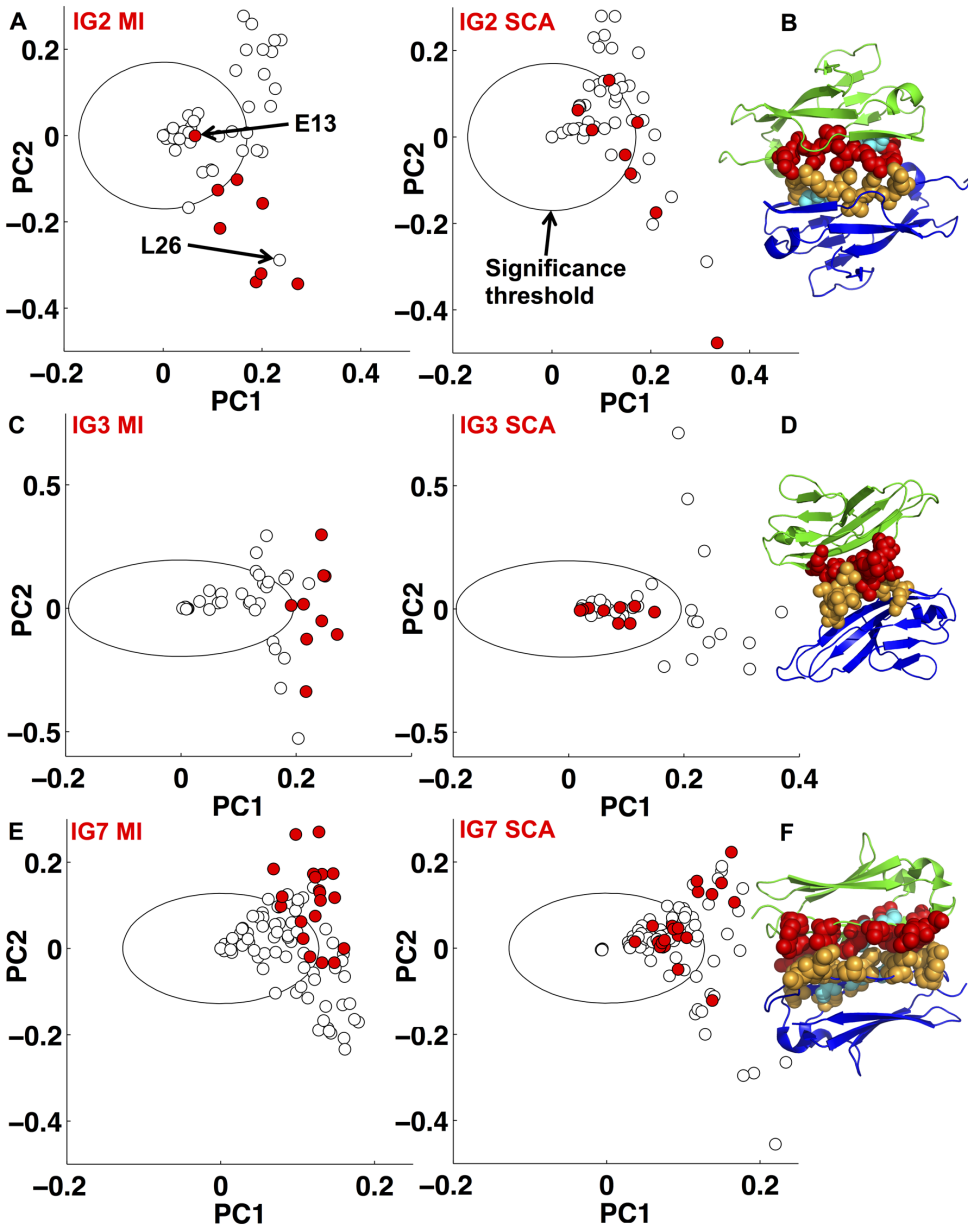
A) PCA of the correlation matrix produced by (left) MI (weighting function *w*_var_ and 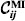) and (right) SCA (weighting function *w*_cons_ and 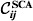) applied to the Dscam A) Ig2 C) Ig3, and E) Ig7 domain alignments. The plots produced by MI analysis in each of A, C and E largely recover the experimentally verified residues (red) identified in [24], [25]. B), D), F) Experimentally verified residues (red in monomer one, and orange in monomer two) mapped onto the corresponding domain dimer interface from crystal structure 3DMK [26]. Those residues identified in [24], [25] that fall inside our threshold circle on the MI plots are colored cyan in B and F. The calibration of the circle radii in these plots are explained in the methods section.

In Fig. 4 we show the results of these analyses (see also Figs. S5–7 in file S1). On the left we use the weighting function 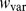 to analyze each of the three alignments, while on the right we apply 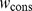 to the same alignments. Residues that were shown experimentally (Ig2, Ig3) or inferred from crystal structure data (Ig7) to determine interaction specificity are colored red. Note that these residues are grouped together and separated from the bulk when 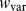 is used, but this is not the case when 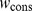 is used.

We note that Ig2 residue E13, part of the beta strand shown to determine specificity, is not identified by the algorithm with 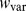 (Fig. 4A). Indeed, inspection of the crystal structure reveals that the side chain of this residue faces away from the Ig2 dimer interface (Fig. 4B). Within *Drosophila melanogaster*, E13 is conserved in ten of the twelve Ig2 sequences suggesting it may not contribute strongly to determining interaction specificity. In contrast, residue L26 clusters with the residues shown to determine interaction specificity (Fig. 4A), yet was not a member of the beta strand shown experimentally to determine specificity. In the crystal structure the side chain of residue 26 makes contact with the equivalent residue across the dimer interface, supporting our prediction that it may play a role in determining interaction specificity. For Ig3 our analysis identifies the experimentally tested residues (Fig. 4B). In the case of Ig7 (Fig. 4E), where 17 specificity-determining-residues were predicted from the crystal structure, our analysis based on 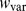 predicts that 14 of these residues are key for specificity determination, and the remaining three residues are close to our threshold.

**Figure 5:**
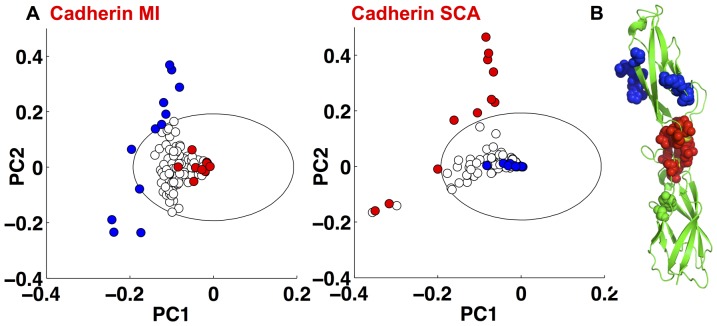
A) PCA of the correlation matrix produced by (left) *w*_var_ and 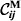, and (right) *w*_cons_ and 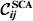 applied to the Cadherin alignment. B) Residues identified by *w*_cons_ and 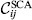 and colored as in (A) shown on crystal structure 1EDH, note that the red sector residues form the calcium binding site; calcium ions colored yellow [29].

In a further example we use both weighting functions to analyze an alignment containing 7829 classical Cadherin domain sequences, members of the Cadherin superfamily [28]. The variable weighting function 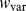 identifies a set of 12 residues, 11 of which are surface exposed (pdbID 1EDH) [29], and hence likely more variable. In contrast 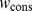 identifies 13 residues (Fig. 5); 11 located in the highly conserved calcium binding domain (red spheres), of which 10 bind calcium ions. Indeed, it was recently shown that while the majority of Cadherin domains have a canonical calcium binding motif, calcium-free Cadherin domains are necessary, for example to allow *Drosophila* N-Cadherin to assume the kinked orientation necessary to fit into the invertebrate intercellular space [30]. Bioinformatic analysis [30] found that Cadherin domains that lack the calcium binding motif make up around 10% of all Cadherin domains. The fact that the weighted algorithm identifies residues involved in calcium binding and the unweighted algorithm identifies surface exposed residues supports our proposal that the weighting identifies correlated residues that are highly conserved.

For our final example we construct an alignment of Protocadherin (Pcdh) domains, for which those residues that determine interaction specificity have not yet been identified. Protocadherins are the largest group in the Cadherin superfamily, and in vertebrates there are multiple isoforms of the clustered *Pcdh-* 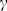 gene. It has been shown experimentally that individual neurons express distinct repertoires of 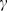-Pcdh isoforms [31], and that these isoforms homodimerize specifically across the cell-cell interface [32]. The specificity is dictated by the EC2 and EC3 domains alone, independently of each other [32]. We used the sequenced genomes of vertebrate species to construct alignments of just over 1000 sequences for each of 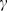-Pcdh domains EC1–4. Our assumption is that the specificity determining residues are highly variable so we use 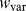 to identify putative specificity determining residues.

**Figure 6:**
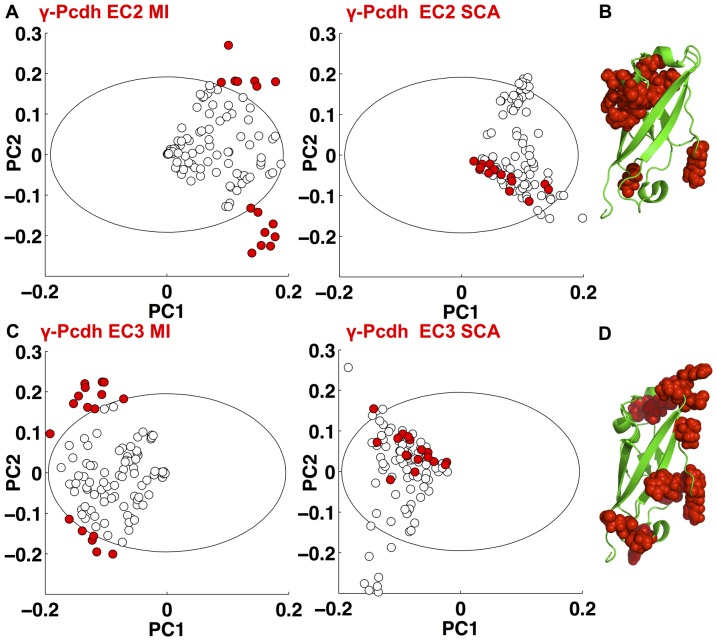
A) PCA of the correlation matrix produced by (left) *w*_var_ and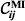 and (right) *w*_cons_ and 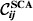, applied to the Pcdh-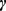 EC2 alignment. B) Residues identified and colored as in (A) mapped onto the crystal structure 1WUZ of Pcdh-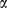 [33]. C) PCA of the correlation matrix produced by (left) *w*_var_ and 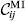, and (right) *w*_cons_ and 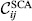 applied to the Pcdh-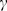 EC3 alignment. D) Residues identified and colored as in (B) mapped onto the crystal structure 1WUZ of Pcdh-*α* [33].

The results of our analysis are shown in Fig. 6. We identify small and largely distinct sets of residues within domains EC2 and EC3. Mapping these residues onto the only solved crystal structure of a Pcdh domain, *Pcdh-α* [33], reveals that they are surface exposed, supporting our suggestion that at least some of these residues may play a role in interaction specificity, as found for the Dscam domains. In addition our analysis of domains EC1 and EC4, which were experimentally shown not to determine interaction specificity, highlights largely distinct sets of residues from the analysis of the EC2 and EC3 domains (Fig. S8 in file S1).

## Discussion

In this manuscript we compare two experimentally verified algorithms for detecting phenotype-controlling residues from a multiple sequence alignment, and observe that the performance of the algorithms is alignment specific. We show that the difference occurs because of the different levels of conservation in the phenotype determining residues. We use this observation as the basis for a more general method for detecting phenotype determining residues in sequence alignments. We propose incorporating biological knowledge about the expected conservation level of the phenotype of interest to choose the weighting function: if the phenotype is expected to be highly variable, the weighting function should resemble that used in the analysis of response regulator pairs [7], while if the phenotype is expected to be highly conserved, the weighting function should resemble that used for the serine protease [8].

For a general protein family and phenotype of interest, with some modest knowledge of the relevant phenotypes of sequences in the MSA, a likely scenario for the conservation level of the relevant residues can be formulated, and thus the appropriate weighting function chosen. We demonstrate that this method works both with simulations of artificial sequences and analysis of sequence alignments from Dscam and Cadherin. It is worth noting that the proposed methodology also implies that changing the weighting function used for a single sequence alignment probes the residues responsible for different phenotypes. For example, the residues responsible for structural stability in the response regulator are likely more conserved than those that determine interaction specificity. Thus by using 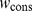, we identify candidates for residues that determine structural stability (See Figs. S9, S10, *Text* S1 in file S1). While we have restricted out attention to the weighting functions used in [7], [8], more generally there is a continuum of possible weighting functions, and a valuable direction for future study is to determine whether there are shapes of weighting functions that give even greater discriminative power.

## Materials and Methods

Statistical tests for detecting pairs of sequence positions in an MSA that do not mutate independently compare amino acid frequencies in each column (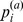 frequency of *a*th amino acid in column *i*) with the distribution of amino acids in column pairs (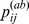 frequency of the pair 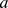 and 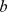 in columns 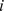 and 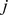 respectively). The results are organized into a matrix of correlation values 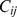. Many metrics for computing 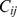 have been proposed (see e.g. [4], [6], [18], [19]). The raw correlation score computed using mutual information (MI) is given by 
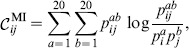
(2)whereas the raw correlation score introduced by Ranganthan and co-workers in SCA [8], [16] is given by 
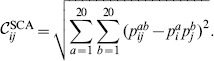
(3)


In [8], Ranganathan and co-workers showed that a simpler formulation of this raw score correlation score produces results that are largely equivalent; here, scores for 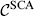 are computed using a binary approximation, in which only the most prevalent amino acid in the MSA is considered. To be consistent with the literature, we use this simpler approximation throughout when computing 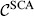; though we note that making a binary approximation is particularly relevant when a column of residues is dominated by a single amino acid, as then it makes sense to distinguish between that residue and all others. When a column is highly variable, the binary approximation is not appropriate, because there are more than two relevant states of the system. For that reason, using a non-binary approximation for 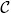, i.e. Eq. (2), does not favor conserved columns over variable ones.

The conservation of column 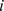 can be measured by the entropy 
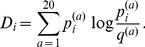
(4)


The weighting function 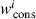 for column 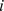 used in SCA, is given by 
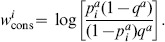
(5)where 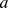 is the most common amino acid in column 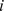. Here 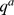 is the background frequency of amino acid 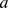 in proteins, corrected for the fraction of gaps occurring in the alignment [8]. This weighting function was motivated by a perturbation analysis of the sequence alignment and previously implemented via a bootstrap procedure [8], [16], [34]. In contrast, [7] did not apply a weighting function to the mutual information scores, which we have described as a constant function 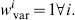
(6)


In [8] principal components analysis (PCA) is used to identify groups of residues that control different phenotypes. The significance threshold circles in Figs. 4, 5 and 6 provide a guide to the null distribution of residue scores that are likely due to finite sample effects, if we assume that the sampling noise is provided by independent draws from identical Gaussian distributions. The radius of this distribution depends on 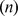, the number of residues in the protein domain, the number of sequences in the alignment and the appropriate noise model. In Fig. 4, partly guided by the experimental data, the radius is 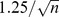 (see Text S1 in File S1 for further details).

## Supporting Information

File S1
**Supporting files.** **Figure S1**, The results of (A) unweighted MI and (B) weighted SCA analyses of the the alignment of serine protease sequences from [8]. The residues colored in red, green and blue are those identified in [8] as being members of intra-protein pairs that have high SCA scores. **Figure S2**, The results of (A) unweighted MI and (B) weighted SCA analyses of the the alignment of concatenated HK-RR cognate pair sequences from [7]. The residues colored in red and green are those identified in [7] as being members of inter-protein pairs that have high MI scores. The red groups contains residues shown experimentally to determine interaction specificity together with residues with high MI scores that are structurally contiguous to the experimentally tested residues. **Figure S3**, A) Weighting function 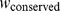 used in [8] to analyze the serine protease family sequence alignment. B) An alternative weighting function, 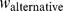, which maximally weights a different range of conservation values to the function in A. C) The sectors for the serine proteases established using t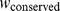 via principal components analysis (PCA). D) PCA applied to the coupling matrix constructed using 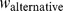. Residues are colored according to the color scheme in C. Note that while the blue and red sectors are largely recovered with this analysis, the green sector, which defines the catalytic heart of the protein, is not. **Figure S4**, Simulations of molecular evolution in which correlated residues evolve at a different rate to uncorrelated residues. A) Correlated residues are more conserved, the correlated mutation rate is 0.06 while the uncorrelated mutation rate 0.12. B) Correlated residues are less conserved, the correlated mutation rate 0.06 and the uncorrelated mutation rate 0.03. The histograms on the left show the distribution of scores attained by the SCA algorithm, consisting of weighting function 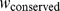 and 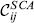, while the right panel shows the distribution of scores attained by applying the MI algorithm, consisting of weighting function 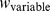 and 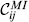, to the same data. These simulations find that SCA is able to detect correlated pairs with greater reliability when they are more conserved than uncorrelated pairs, while the reverse is true of MI. **Figure S5**, MI and SCA analyses of the Dscam Ig2 domain alignment. Those amino acids that were experimentally shown to be involved in determining homodimerization specificity in [24] are colored in red. The circle of radius 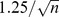, where 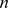 is the number of aligned residues, indicates the extent of points that might occur due to noise under a null hypothesis. **Figure S6**, MI and SCA analyses of the Dscam Ig3 domain alignment. Those amino acids that were experimentally shown to be involved in determining homodimerization specificity in [24] are colored in red. The circle of radius 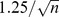, where 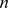 is the number of aligned residues, indicates the extent of points that might occur due to noise under a null hypothesis. **Figure S7**, MI and SCA analyses of the Dscam Ig7 domain alignment. Those amino acids that were inferred based on their structural locations to be involved in determining homodimerization specificity in [26] are colored in red. The circle of radius 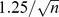, where 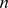 is the number of aligned residues, indicates the extent of points that might occur due to noise under a null hypothesis. **Figure S8**, MI analysis of 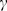-Pcdh domains (A) EC1 and (B) EC4. The circle of radius 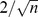, where 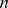 is the number of aligned residues, indicates the extent of points that might occur due to noise under a null hypothesis. The amino acids that lie outside the threshold circle in the PC1-PC2 plot are colored in red on the structure 1WUZ of the homologous Pcdh-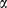 [33]. **Figure S9**, The results of (A) MI, consisting of weighting function 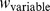 and 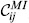, and (B) SCA, consisting of weighting function 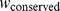 and 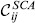 analyses of the the alignment of concatenated HK-RR cognate pair sequences from [7], as in Fig. S2 in file S1. However, here the residues are colored according to their position in the SCA PC2-PC3 plot, these principal components were chosen arbitrarily from combinations of the top few principal components, note that largely the same residues would be chosen using the other PC combinations. **Figure S10**, Analysis of the serene protease domain alignment using (A) MI, consisting of weighting function 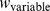 and 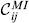, and (B) SCA, consisting of weighting function 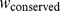 and 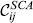. In each case a group of residues that includes members of the S1 substrate binding pocket (purple dots) and the L1 (blue dots) and L2 (light blue dots) selectivity determining loops is identified by the algorithm indicated. Here we compare the groups of residues identified by each algorithm by showing them as solid spheres on the experimentally determined crystal structure 1YF4 [35] of trypsin (green cartoon) in complex with the inhibitor peptide vasopressin (shown as dark blue sticks). Those residues shown in red are identified by MI (A) or SCA (B) but are not part of the S1/L1/L2 features. C) Analysis of the HK-RR domains. The yellow sector, colored as in Fig. S3 in file S1 on crystal structure 1F51 [36], which avoids the N-terminal helix (see supplementary text). This sector is identified largely intact by both the weighted SCA and the unweighted MI analysis of the HK-RR alignment. **Text S1**.(PDF)

Sequence Alignments S1 **Sequence alignments built for this work and analysed in the main text.**
(ZIP)
