# Supplementary material for: Conservation Weighting Functions Enable Covariance Analyses to Detect Functionally Important Amino Acids
Source: PLoS One. 2014 Nov 7;9(11):e107723. doi: 10.1371/journal.pone.0107723 (PMC4224327; doi:10.1371/journal.pone.0107723)
Supplement: File S1 — Supporting files. Figure S1, The results of (A) unweighted MI and (B) weighted SCA analyses of the the alignment of serine protease sequences from [8]. The residues colored in red, green and blue are those identified in [8] as being members of intra-protein pairs that have high SCA scores. Figure S2, The results of (A) unweighted MI and (B) weighted SCA analyses of the the alignment of concatenated HK-RR cognate pair sequences from [7]. The residues colored in red and green are those identified in [7] as being members of inter-protein pairs that have high MI scores. The red groups contains residues shown experimentally to determine interaction specificity together with residues with high MI scores that are structurally contiguous to the experimentally tested residues. Figure S3, A) Weighting function used in [8] to analyze the serine protease family sequence alignment. B) An alternative weighting function, , which maximally weights a different range of conservation values to the function in A. C) The sectors for the serine proteases established using t via principal components analysis (PCA). D) PCA applied to the coupling matrix constructed using . Residues are colored according to the color scheme in C. Note that while the blue and red sectors are largely recovered with this analysis, the green sector, which defines the catalytic heart of the protein, is not. Figure S4, Simulations of molecular evolution in which correlated residues evolve at a different rate to uncorrelated residues. A) Correlated residues are more conserved, the correlated mutation rate is 0.06 while the uncorrelated mutation rate 0.12. B) Correlated residues are less conserved, the correlated mutation rate 0.06 and the uncorrelated mutation rate 0.03. The histograms on the left show the distribution of scores attained by the SCA algorithm, consisting of weighting function and , while the right panel shows the distribution of scores attained by applying the MI algorithm, consisting of weighting [file pone.0107723.s001.pdf]

SUPPLEMENTAL MATERIALS AND METHODS

Algorithms and Weighting Functions

The Statistical Coupling Analysis (SCA), introduced by Ranganathan and co-workers, involves weighting the raw correlation scores of pairs of residues by a weight that is derived from the conservation level of each residue in the pair [2, 5]. This function is plotted in Fig. S1A, and does not depend on the alignment or protein phenotype of interest. This raises the question of whether the sectors identified in Fig. 1A are robust to changing this function. We find that most sector residues are lost when no weighting function is used (Figure 1C), although some of the blue sector, which determines the protein's stability, is recovered. In addition, Figs. S1B, D show that moving the maximum of the weighting function changes the ability to detect each of the three sectors, supporting the conclusion that the residue composition of sectors is sensitive to which weighting function is used.

In the main text we restrict the discussion to four alternatives that are directly motivated by previous work [2, 8]. These are constructed from the two statistical methods explored in these papers, $\mathcal{C}_{ij}^{\mathrm{SCA}}$ and $\mathcal{C}_{ij}^{\mathrm{MI}}$ combined with the weighting functions w_{cons}^{i} used in [2] and w_{var}^{i} used in [8]. Taking all combinations of statistical method and weighting function results in the four algorithms analyzed in the text, which are described by the following equations.

$$\text{Weighted Mutual Information: } C_{ij} = w_{\mathrm{cons}}^{i} w_{\mathrm{cons}}^{j} \mathcal{C}_{ij}^{\mathrm{MI}} = w_{\mathrm{cons}}^{i} w_{\mathrm{cons}}^{j} \sum_{a=1}^{20}\sum_{b=1}^{20} p_{ij}^{ab} \log \frac{p_{ij}^{ab}}{p_i^a p_j^b}, \quad (1)$$

$$\text{Mutual Information: } C_{ij} = w_{\mathrm{var}}^{i} w_{\mathrm{var}}^{j} \mathcal{C}_{ij}^{\mathrm{MI}} = w_{\mathrm{var}}^{i} w_{\mathrm{var}}^{j} \sum_{a=1}^{20}\sum_{b=1}^{20} p_{ij}^{ab} \log \frac{p_{ij}^{ab}}{p_i^a p_j^b}, \quad (2)$$

$$\text{Un-Weighted Statistical Coupling Analysis: } C_{ij} = w_{\mathrm{var}}^{i} w_{\mathrm{var}}^{j} \mathcal{C}_{ij}^{\mathrm{SCA}} = w_{\mathrm{var}}^{i} w_{\mathrm{var}}^{j} \sqrt{\sum_{a=1}^{20}\sum_{b=1}^{20} (p_{ij}^{ab} - p_i^a p_j^b)^2} \quad (3)$$

$$\text{Statistical Coupling Analysis: } C_{ij} = w_{\mathrm{cons}}^{i} w_{\mathrm{cons}}^{j} \mathcal{C}_{ij}^{\mathrm{SCA}} = w_{\mathrm{cons}}^{i} w_{\mathrm{cons}}^{j} \sqrt{\sum_{a=1}^{20}\sum_{b=1}^{20} (p_{ij}^{ab} - p_i^a p_j^b)^2} \quad (4)$$

In general it is hard for more conserved residues to attain a high correlation score, since they change less frequently in the sequence alignment so less information is available about how correlated they are. The absence of a weighting function thus effectively attaches more weight to the correlation scores of residues that are highly variable. A simple method for dealing with this effect is to normalize each column to have unit variance. The particular weighting function, $w_{\mathrm{conserved}}$, used in [2] attaches even more weight to conserved residues than this simple normalization.

We note from Fig. 1 and Fig. S3 that the green sector is particularly sensitive to the choice of weighting function. This result makes sense, since the green sector contains the catalytic triad, which is universally conserved amongst serine proteases, and is only detected because the serine protease alignment contains a small number of related proteins that have lost catalytic activity. These include 13 haptoglobin sequences, evolutionarily related proteins that bind hemoglobin and lack proteolytic activity. Any coupling signal from a pair of highly conserved residues is necessarily rather faint as there is less information (mutations) available from which to detect coupling. This explains why a weighting function similar to $w_{\mathrm{conserved}}$ is necessary to detect the green sector.

To illustrate our approach in a specific example we turn to the blue sector, involved in stabilizing the folded protein [2]. Halabi *et al.* observe that grouping the sequences by homology of residues in the blue sector separates proteins from vertebrates and invertebrates. Just over half the sequences in the alignment are from vertebrate organisms. Thus if a residue were to perfectly distinguish between vertebrate and invertebrate proteins, it would be roughly

50% conserved. Hence the optimal strategy for detecting such sites is to weight the coupling score by a function that attains its maximal value around a conservation level of 50% (Fig S3B is an example though many functions fulfill this condition). Figure S3D shows the result of applying PCA to the correlation matrix generated by using the weighting function in Fig S3D to weight the SCA analysis. Note that the green sector is no longer detected by the analysis, while the red and blue sectors are recovered largely intact, though some additional residues group with these sectors.

Detecting Groups of Residues

A phenotype determined by more than two residues will lead to higher order correlations in the sequence alignment. In many cases this will result in a group of residues that are all pairwise correlated, enabling the matrix of pairwise correlation values to reveal higher order correlations [2, 8, 10]. A single pair of residues might randomly be correlated due, for example, to phylogeny. However, it is considerably less likely for a group of several residues to have above background pair-wise coupling scores by chance. Therefore, the signal-to-noise ratio can be vastly improved by looking for groups of residues that are all pairwise correlated with each other. This reduces the resolution of an algorithm, as individual pairs that are coupled to each other, but not to other positions, will not be detected. Yet in many cases more than two residues will be involved in maintaining the phenotypic property of interest.

One unsupervised method of detecting this structure, used in [2], is to perform PCA. PCA transforms a number of correlated variables into a smaller number of uncorrelated variables called principal components, ordered according to their eigenvalues, i.e. amount of variability that each explains. The structure of the correlation matrix dictates the number of significant principal components. Plotting these components against each other reveals groups of residues that have significant eigenvector coefficients, implying that these residues co-vary, at least to some extent, as groups.

In order to identify residues that have significant eigenvector coefficients, it is necessary to have a null model for the eigenvector coefficients that would be expected if there were no amino acid pairs that were correlated beyond the pairwise correlations expected from descent from a single ancestral sequence. Because the analysis is carried out with a finite number of observations (sequences) there will be noise in the correlation measurements. If we assume that the sampling noise is provided by independent draws from identical Gaussian distributions, then we would expect the eigenvector coefficients to be drawn from a Gaussian distribution with mean zero and variance $1/\sqrt{n}$ where n is the number of sequence residues. To be conservative, we have regarded eigenvector coefficients to be significant if they are larger than $2/\sqrt{n}$ (i.e. two standard deviations) for the Cadherin alignments, and $1.25/\sqrt{n}$ for the Dscam alignments. The threshold was changed for the Dscam alignments because of the small number of sequences in these alignments. While these assumptions are reasonable further experimental data will better inform our thinking about the type of noise that is present.

The analysis in [2] goes a step further than this and uses PCA to identify groups of residues that co-vary independently of each other, and also control distinct phenotypes within the same protein. While it is possible that applying this analysis to the Dscam, Cadherin and Protocadherin datasets might reveal groups of residues responsible for different protein properties, a lack of experimental data prevents us from testing this hypothesis. We note that a similar analysis was not carried out in [8]; in the section 'Analysis of HK-RR dataset' below we explore the application of this methodology to the HK-RR dataset, and speculate about the possible phenotype that one group of residues might control.

Simulation methods

In these simulations most amino acids evolve independently through a Markov model whose mutation matrix is derived from BLOSUM90 [4]. Specifically, we assume that the sequence consists of 100 sites that follow processes that are Markovian independent, identically distributed and constant in time. For a fixed site $\mathbf{P}(t)$ is the vector of state probabilities at time t, then

$$\mathbf{P}'(t) = Q\mathbf{P}(t)$$

where Q is the matrix describing the transition rates between the different possible states. In our simulation, the possible states are the 20 amino acids, and the transition rate matrix Q is given by the BLOSUM90 matrix.

The correlations are enforced by requiring that if the first residue of a pair mutates, then the second also mutates; the second residue of each pair is not given the opportunity to mutate alone. We first explore whether the performance of each algorithm depends on the characteristics of the alignments generated. We vary two alignment properties: the average mutation rate and the phylogenetic tree according to which the sequences are generated, which is parameterized by the number of duplication (branching) events that occur (Fig. 3A). For each set of parameters (mutation rate, # branching events) we simulate ten rounds of replication to generate an alignment of $N = 2^{10}$ sequences, and ask whether the correlated mutation algorithms analyzed above can discriminate between those residues constrained to be correlated and those that evolve independently. Each sequence in the alignment has $M = 100$ residues, of which $Q = 20$ are constrained to mutate as ten pairs leaving 4940 pairs to mutate independently.

We determine how successful each algorithm is at finding the correlated pairs using the following procedure. A correlation score is computed for each pair of residues in the alignment. We then divide all $M(M-1)/2$ distinct residue pairs into two groups; those which mutate independently and those pairs whose evolution is constrained. For each set, we obtain a distribution of correlation scores. The algorithm is able to discriminate perfectly between these two sets if the two distributions of correlation scores do not overlap. As there are many more uncoupled pairs than coupled pairs, statistically it will be difficult to detect the coupled pairs if the distributions overlap substantially. To quantify discrimination ability, we define a metric by dividing the lowest score assigned to a correlated pair by the highest score assigned to a pair that mutates independently.

Analysis of the serine protease dataset

As discussed in the main text, we applied MI, , consisting of weighting function w_{variable} and $\mathcal{C}_{ij}^{MI}$, to the alignment of serine protease residues from [2]. The residues identified by SCA (Fig. S1), and experimentally shown to control different phenotypes, are largely not identified by MI. In particular no member of the green sector (catalytic triad and associated residues), is identified by any of the MI principal component plots. This immediately raises the question of which residues the unweighted MI analysis identifies in this dataset and, perhaps more pertinently, what phenotypes those residues may influence. If we make the assumption that those residues that control substrate specificity mutate when the specificity changes, then because this alignment contains proteins with at least six different substrate specificities, we would expect an algorithm that up-weights residues that are poorly conserved to detect residues that determine substrate specificity.

Given this hypothesis, it is interesting that there is significant overlap between the set of residues identified by MI with those identified by SCA, consisting of $w_{\text{conserved}}$ and $\mathcal{C}_{ij}^{SCA}$, and shown to determine substrate specificity (nine shared residues, out of 22 total distinct residues identified by either algorithm). This fact, taken together with the fact that there are at least 6 different substrate specificities well represented in the alignment, suggests that

identification of specificity determining residues may be improved by using an intermediate weighting function. We note that applying MI to the serine protease alignment from [2] highlights residues in the S1 pocket and the L1 and L2 loops that were not identified by SCA, but which are known to be involved in determining substrate specificity [3] (Fig. S10A, B). Specifically of the 29 residues that make up the S1 pocket and L1/L2 loops, 7 are identified by both algorithms, 5 are identified by only SCA, while 6 are identified by only MI. In addition MI identifies two pairs of contacting residues, each of which make contact with the S1 pocket suggesting they could play a role in determining substrate specificity. These predictions would benefit from experimental evaluation.

Analysis of HK-RR dataset

Repeating the mutual information (MI) analysis of the HK-RR sequence alignment carried out in [8], as discussed in the text, demonstrates that the residues that participate in those inter-protein pairs with the highest MI scores are identified by the principal component plots (red, and green Fig. S2. Those histidine kinase (HK) residues that were experimentally tested in [8], and the regulator (RR) residues identified in [8] that are structurally adjacent are colored red. The remaining residues identified in [8] are colored green. In contrast, using SCA ($w_{\text{conserved}}$ and $\mathcal{C}_{ij}^{SCA}$) to analyze the same alignment produces the results shown in Fig. S2. Note that those residues identified in [8] and colored do not score significantly in this analysis.

Based on the findings in [2], residues that are both correlated and well-conserved are likely to be involved in maintaining structural stability or controlling catalytic activity. If we use the weighted SCA results to identify residues, and following the methodology in [2] color those residues identified blue, cyan and yellow (Fig. S9, PCs 2 and 3), we find many residues that are not identified by the unweighted MI analysis. However, the sets of residues identified by each algorithm are not mutually exclusive. In particular the yellow sector identified by SCA is identified largely intact by the unweighted MI analysis. In Fig. S10C these residues are mapped onto crystal structure 1F51 - they form a network of residues within the response regulator that are close in 3D structure but avoid the key interface involving the N-terminal end of the first helix in the response regulator which has been shown to determine interaction specificity [1]. We speculate that the network of yellow residues could control the structural stability of the response regulator. Indeed, if a pair of residues has a high raw correlation score, while both members have intermediate conservation level, the scores under either a weighted or unweighted algorithm could be large enough to warrant identification in both cases.

[1] Bell, C. H., Porter, S. L., Strawson, A., Stuart, D. I. and Armitage, J. P. (2010). Using structural information to change the phosphotransfer specificity of a two-component chemotaxis signalling complex. PLoS biology *8*, e1000306.

[2] Halabi, N., Rivoire, O., Leibler, S. and Ranganathan, R. (2009). Protein sectors: evolutionary units of three-dimensional structure. Cell *138*, 774–786.

[3] Hedstrom, L., Szilagyi, L. and Rutter, W. J. (1992). Converting trypsin to chymotrypsin: the role of surface loops. Science *255*, 1249–1253.

[4] Henikoff, S. and Henikoff, J. G. (1993). Performance evaluation of amino acid substitution matrices. Proteins: Structure, Function, and Bioinformatics *17*, 49–61.

[5] Lockless, S. W. and Ranganathan, R. (1999). Evolutionarily conserved pathways of energetic connectivity in protein families. Science *286*, 295–299.

[6] Morishita, H., Umitsu, M., Murata, Y., Shibata, N., Udaka, K., Higuchi, Y., Akutsu, H., Yamaguchi, T., Yagi, T. and Ikegami, T. (2006). Structure of the cadherin-related neuronal receptor/protocadherin-α first extracellular cadherin domain

reveals diversity across cadherin families. Journal of Biological Chemistry *281*, 33650–33663.

[7] Sawaya, M. R., Wojtowicz, W. M., Andre, I., Qian, B., Wu, W., Baker, D., Eisenberg, D. and Zipursky, S. L. (2008). A double S shape provides the structural basis for the extraordinary binding specificity of Dscam isoforms. Cell *134*, 1007–1018.

[8] Skerker, J. M., Perchuk, B. S., Siryaporn, A., Lubin, E. A., Ashenberg, O., Goulian, M. and Laub, M. T. (2008). Rewiring the specificity of two-component signal transduction systems. Cell *133*, 1043–1054.

[9] Syed Ibrahim, B. and Pattabhi, V. (2005). Trypsin inhibition by a peptide hormone: crystal structure of trypsin–vasopressin complex. Journal of molecular biology *348*, 1191–1198.

[10] White, R. A., Szurmant, H., Hoch, J. A. and Hwa, T. (2007). Features of Protein–Protein Interactions in Two-Component Signaling Deduced from Genomic Libraries. Methods in enzymology *422*, 75–101.

[11] Wojtowicz, W. M., Wu, W., Andre, I., Qian, B., Baker, D. and Zipursky, S. L. (2007). A vast repertoire of Dscam binding specificities arises from modular interactions of variable Ig domains. Cell *130*, 1134–1145.

[12] Zapf, J., Sen, U., Hoch, J. A., Varughese, K. I. et al. (2000). A transient interaction between two phosphorelay proteins trapped in a crystal lattice reveals the mechanism of molecular recognition and phosphotransfer in signal transduction. Structure *8*, 851–862.

SUPPLEMENTAL FIGURES

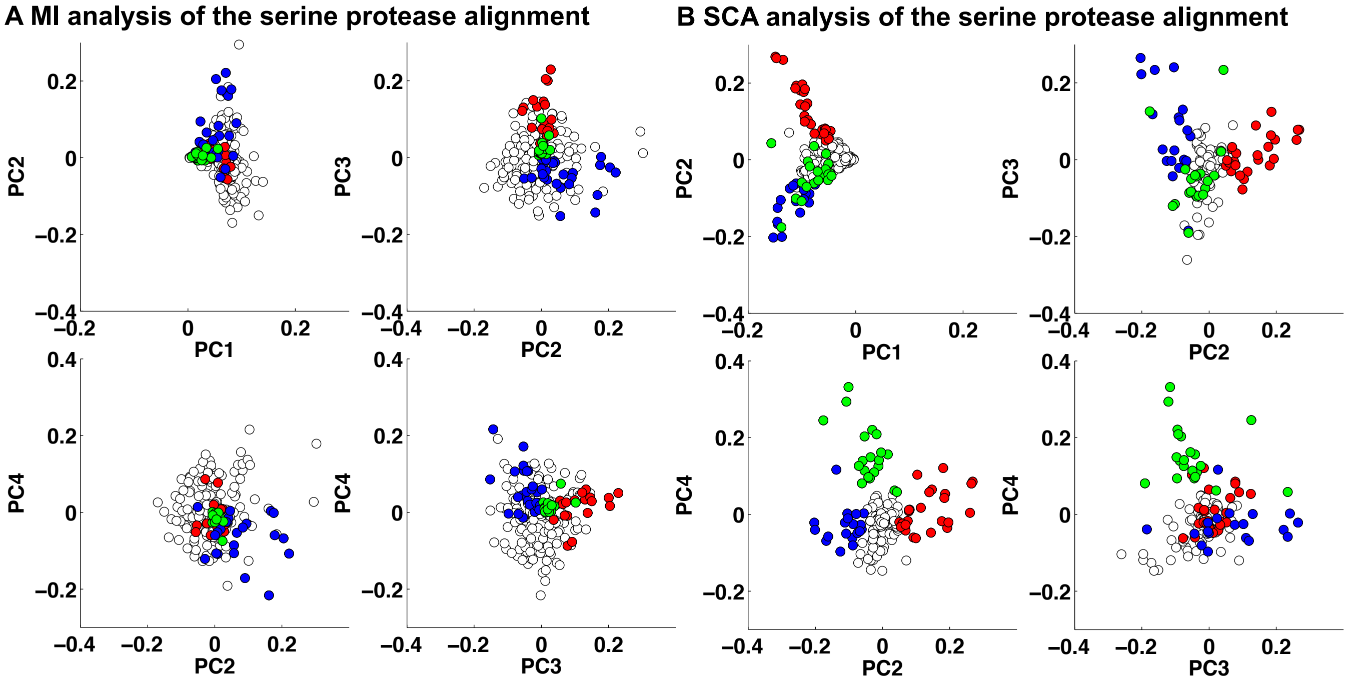

FIG. S1. The results of (A) unweighted MI and (B) weighted SCA analyses of the the alignment of serine protease sequences from [2]. The residues colored in red, green and blue are those identified in [2] as being members of intra-protein pairs that have high SCA scores.

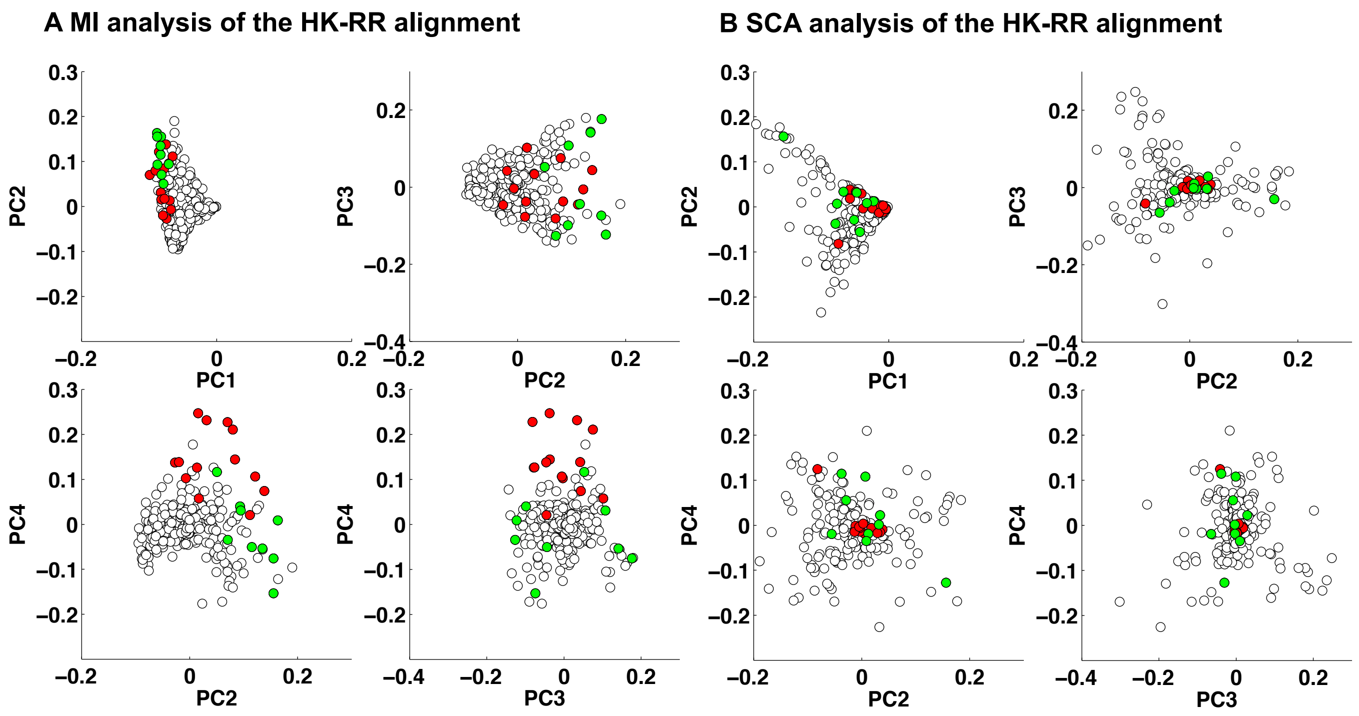

FIG. S2. The results of (A) unweighted MI and (B) weighted SCA analyses of the the alignment of concatenated HK-RR cognate pair sequences from [8]. The residues colored in red and green are those identified in [8] as being members of inter-protein pairs that have high MI scores. The red groups contains residues shown experimentally to determine interaction specificity together with residues with high MI scores that are structurally contiguous to the experimentally tested residues.

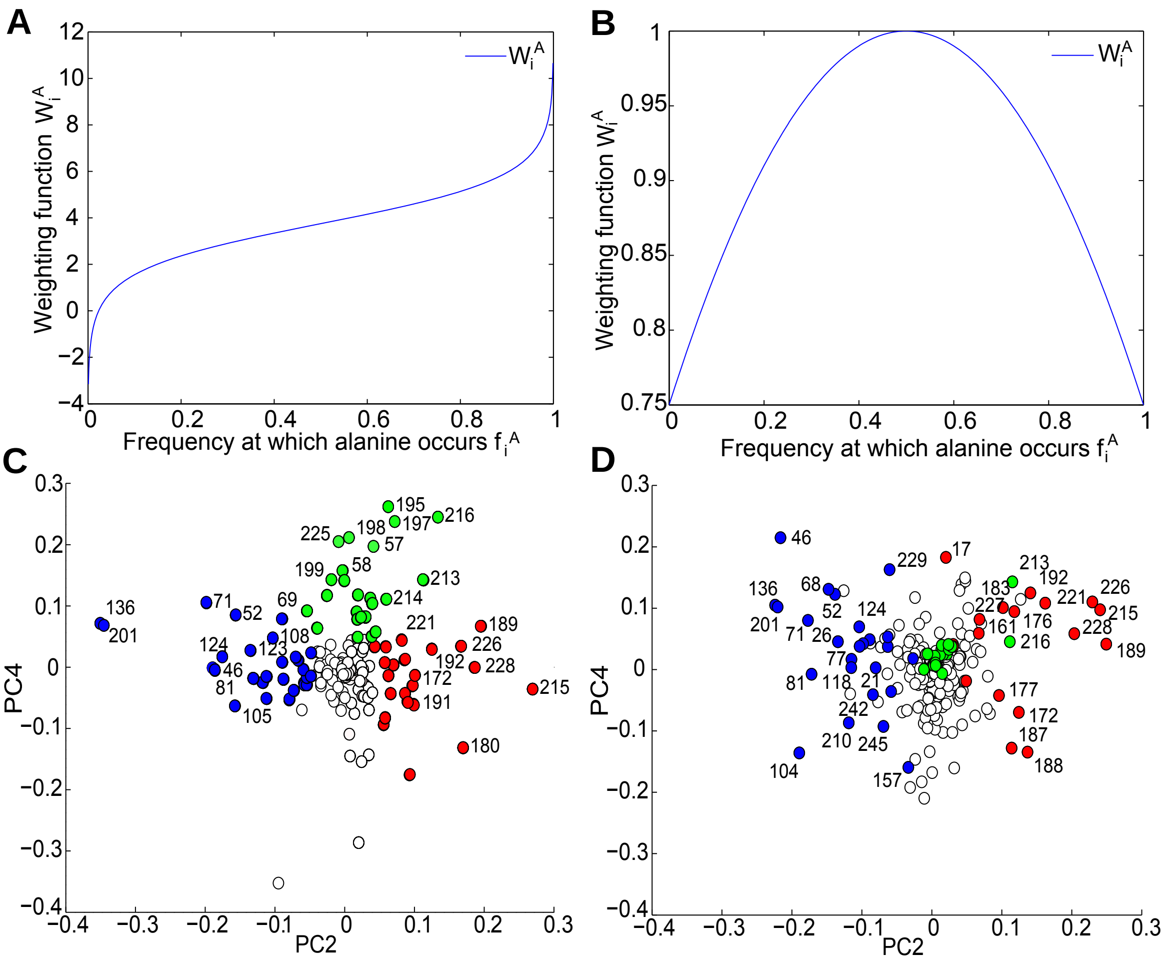

FIG. S3. A) Weighting function $w_{\text{conserved}}$ used in [2] to analyze the serine protease family sequence alignment. B) An alternative weighting function, $w_{\text{alternative}}$, which maximally weights a different range of conservation values to the function in A. C) The sectors for the serine proteases established using t$w_{\text{conserved}}$ via principal components analysis (PCA). D) PCA applied to the coupling matrix constructed using $w_{\text{alternative}}$. Residues are colored according to the color scheme in C. Note that while the blue and red sectors are largely recovered with this analysis, the green sector, which defines the catalytic heart of the protein, is not.

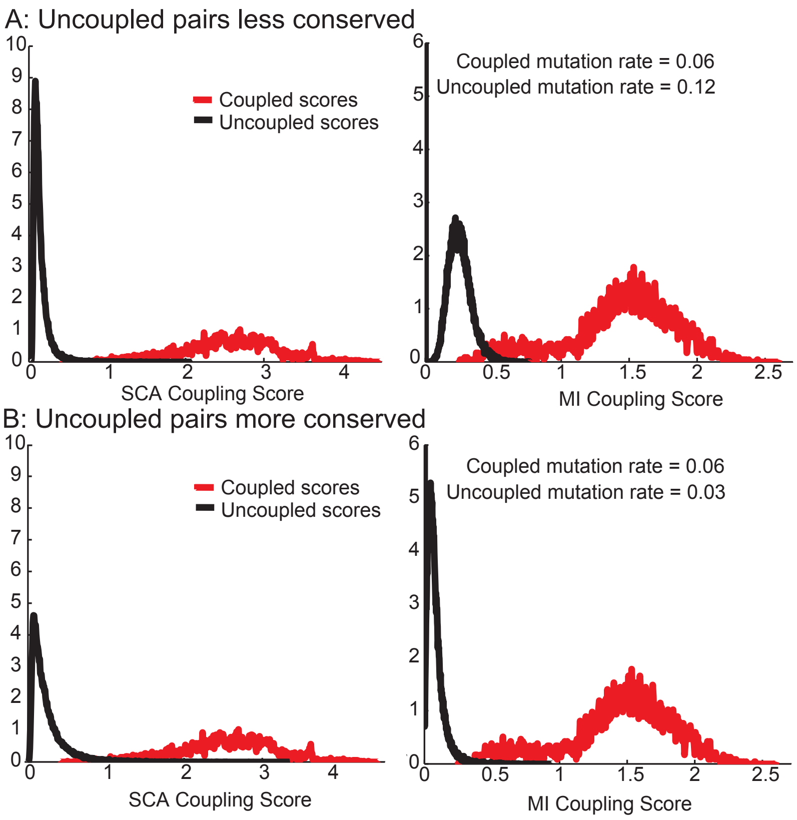

FIG. S4. Simulations of molecular evolution in which correlated residues evolve at a different rate to uncorrelated residues. A) Correlated residues are more conserved, the correlated mutation rate is 0.06 while the uncorrelated mutation rate 0.12. B) Correlated residues are less conserved, the correlated mutation rate 0.06 and the uncorrelated mutation rate 0.03. The histograms on the left show the distribution of scores attained by the SCA algorithm, consisting of weighting function $w_{\text{conserved}}$ and $\mathcal{C}_{ij}^{SCA}$, while the right panel shows the distribution of scores attained by applying the MI algorithm, consisting of weighting function w_{variable} and $\mathcal{C}_{ij}^{MI}$, to the same data. These simulations find that SCA is able to detect correlated pairs with greater reliability when they are more conserved than uncorrelated pairs, while the reverse is true of MI.

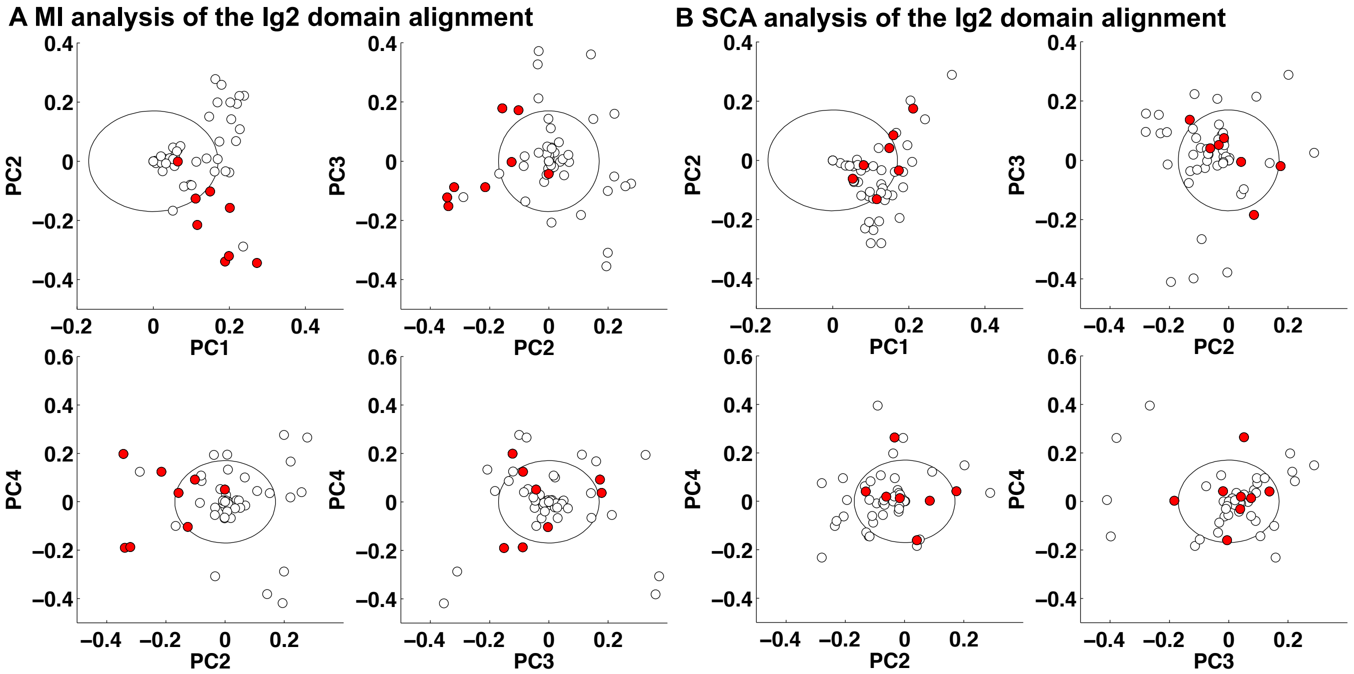

FIG. S5. MI and SCA analyses of the Dscam Ig2 domain alignment. Those amino acids that were experimentally shown to be involved in determining homodimerization specificity in [11] are colored in red. The circle of radius $1.25/\sqrt{n}$, where n is the number of aligned residues, indicates the extent of points that might occur due to noise under a null hypothesis.

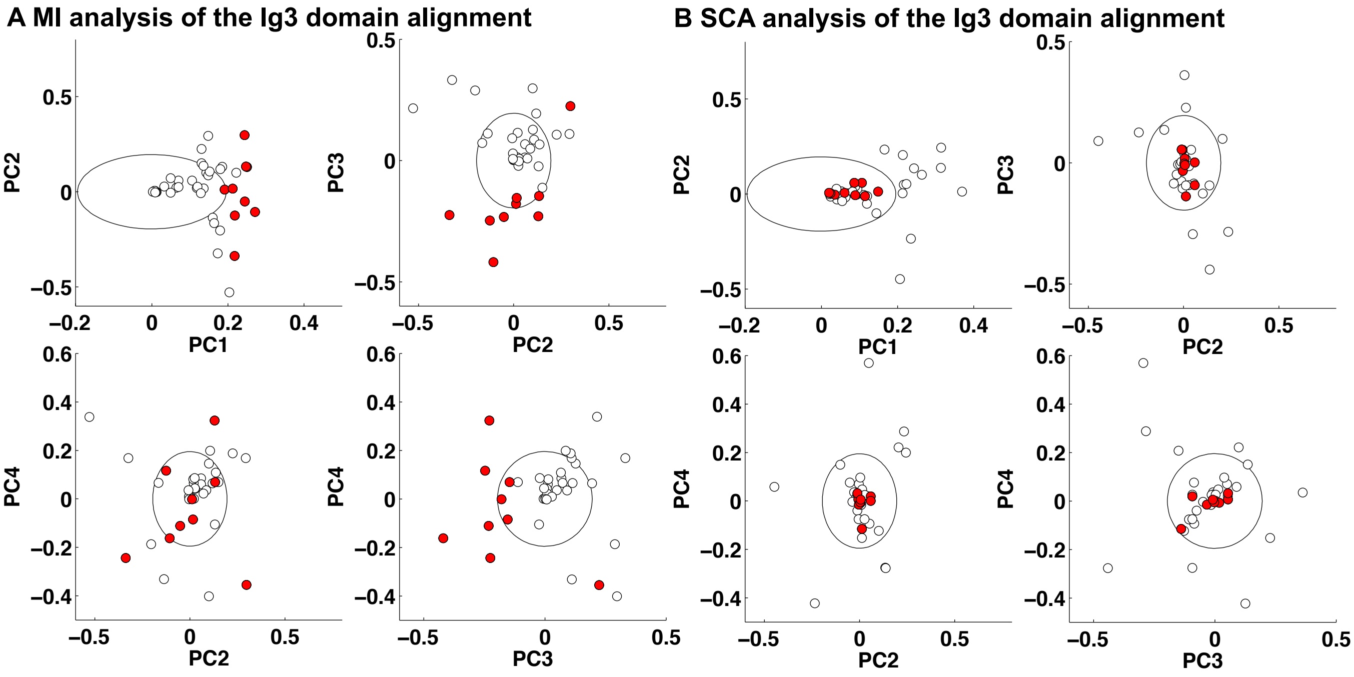

FIG. S6. MI and SCA analyses of the Dscam Ig3 domain alignment. Those amino acids that were experimentally shown to be involved in determining homodimerization specificity in [11] are colored in red. The circle of radius $1.25/\sqrt{n}$, where n is the number of aligned residues, indicates the extent of points that might occur due to noise under a null hypothesis.

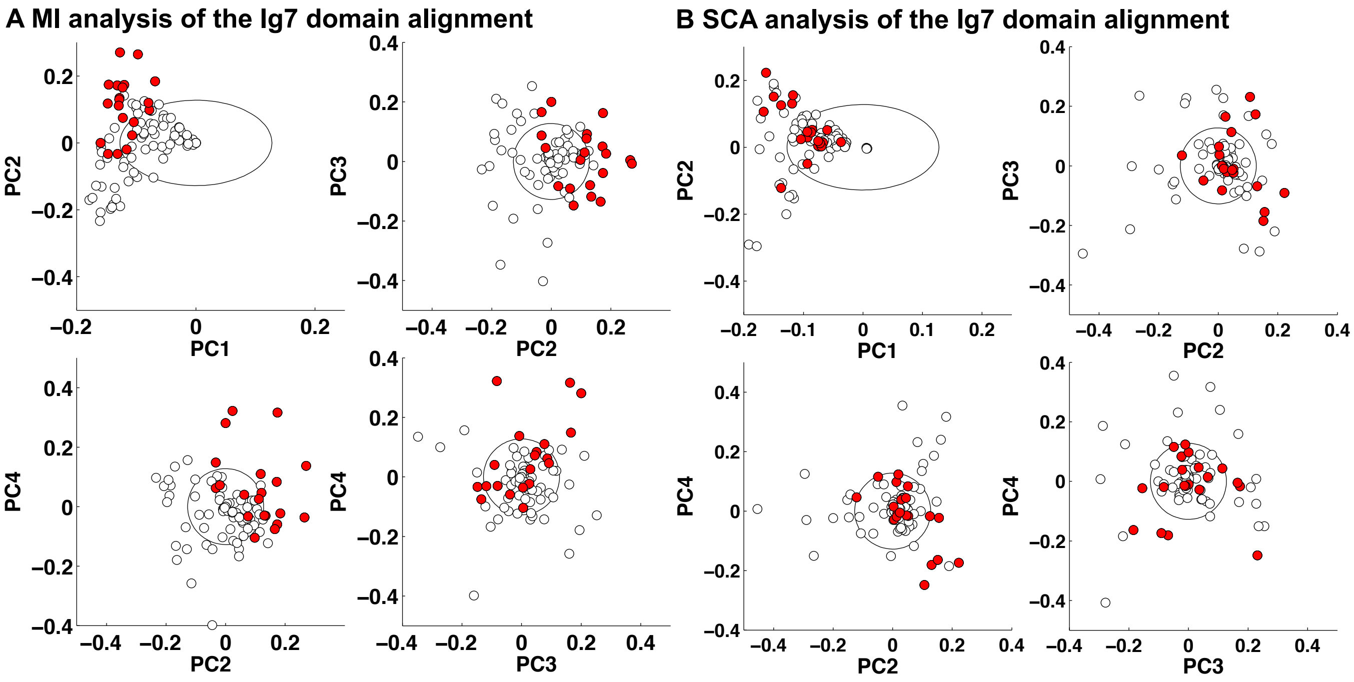

FIG. S7. MI and SCA analyses of the Dscam Ig7 domain alignment. Those amino acids that were inferred based on their structural locations to be involved in determining homodimerization specificity in [7] are colored in red. The circle of radius $1.25/\sqrt{n}$, where n is the number of aligned residues, indicates the extent of points that might occur due to noise under a null hypothesis.

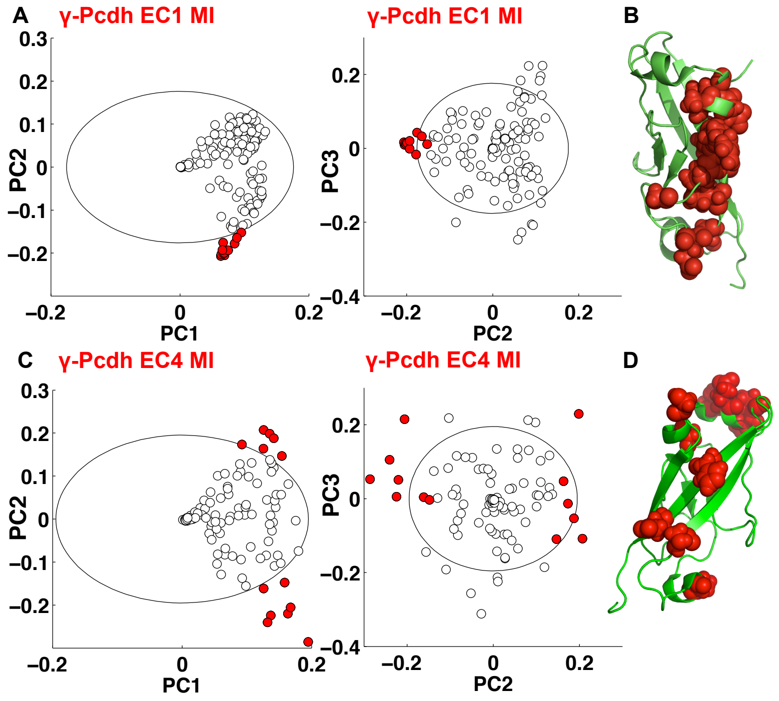

FIG. S8. MI analysis of γ-Pcdh domains (A) EC1 and (B) EC4. The circle of radius $2/\sqrt{n}$, where n is the number of aligned residues, indicates the extent of points that might occur due to noise under a null hypothesis. The amino acids that lie outside the threshold circle in the PC1-PC2 plot are colored in red on the structure 1WUZ of the homologous Pcdh-α [6].

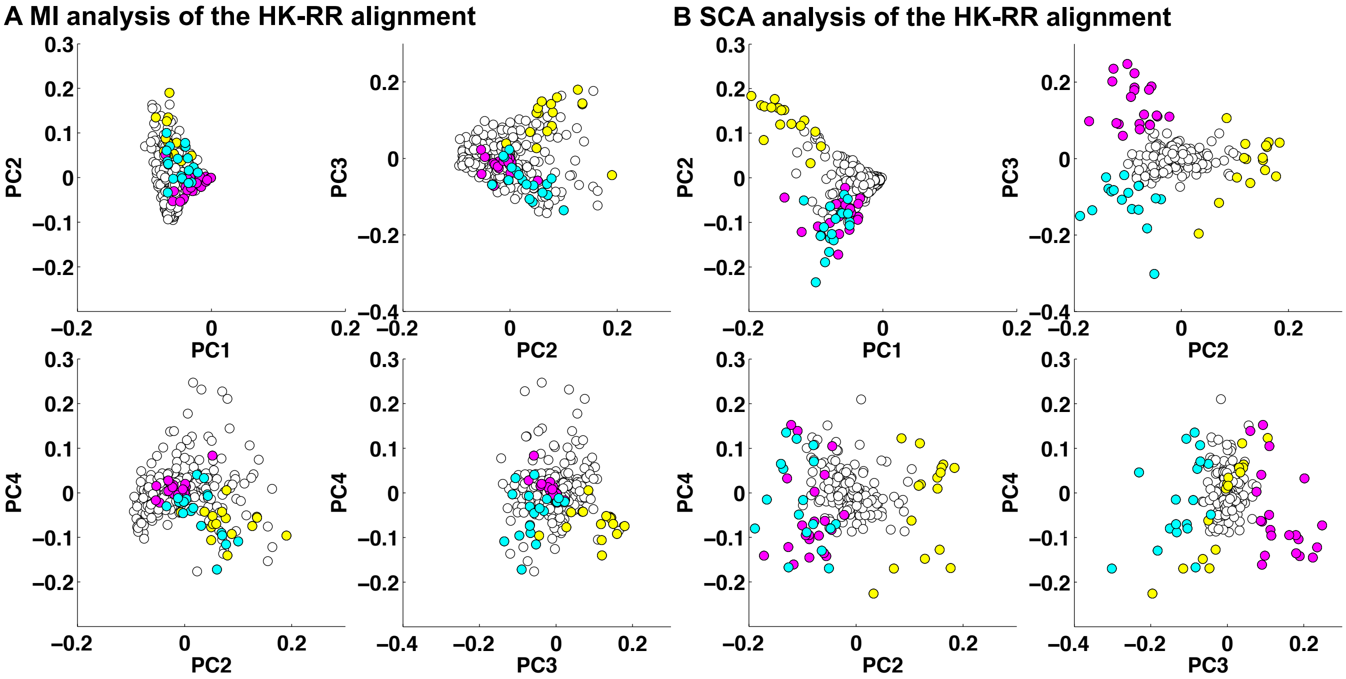

FIG. S9. The results of (A) MI, consisting of weighting function w_{variable} and $\mathcal{C}_{ij}^{MI}$, and (B) SCA, consisting of weighting function $w_{\text{conserved}}$ and $\mathcal{C}_{ij}^{SCA}$ analyses of the the alignment of concatenated HK-RR cognate pair sequences from [8], as in Fig. S2 above. However, here the residues are colored according to their position in the SCA PC2-PC3 plot, these principal components were chosen arbitrarily from combinations of the top few principal components, note that largely the same residues would be chosen using the other PC combinations.

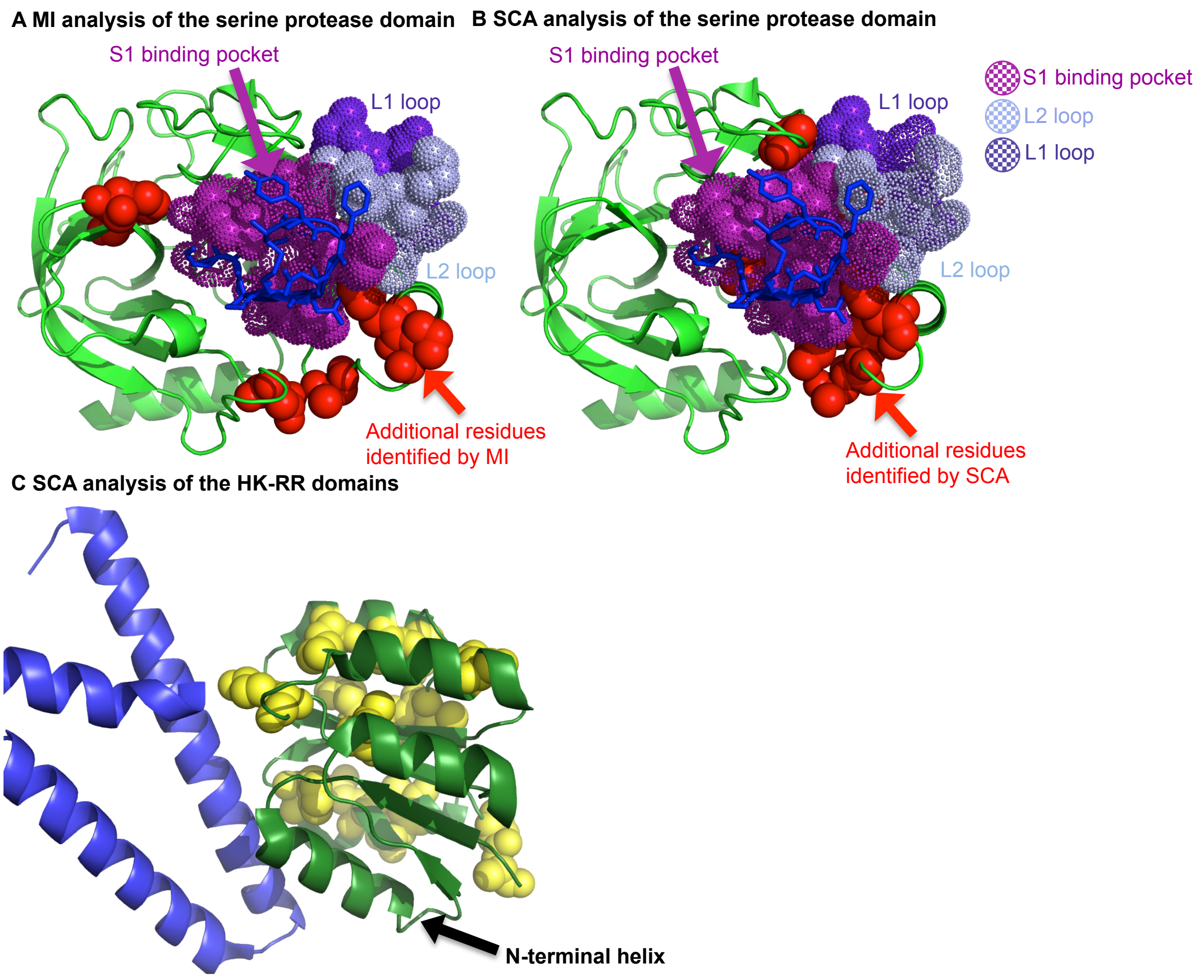

FIG. S10. Analysis of the serene protease domain alignment using (A) MI, consisting of weighting function w_{variable} and $\mathcal{C}_{ij}^{MI}$, and (B) SCA, consisting of weighting function $w_{\text{conserved}}$ and $\mathcal{C}_{ij}^{SCA}$. In each case a group of residues that includes members of the S1 substrate binding pocket (purple dots) and the L1 (blue dots) and L2 (light blue dots) selectivity determining loops is identified by the algorithm indicated. Here we compare the groups of residues identified by each algorithm by showing them as solid spheres on the experimentally determined crystal structure 1YF4 [9] of trypsin (green cartoon) in complex with the inhibitor peptide vasopressin (shown as dark blue sticks). Those residues shown in red are identified by MI (A) or SCA (B) but are not part of the S1/L1/L2 features. C) Analysis of the HK-RR domains. The yellow sector, colored as in Fig. S3 on crystal structure 1F51 [12], which avoids the N-terminal helix (see supplementary text). This sector is identified largely intact by both the weighted SCA and the unweighted MI analysis of the HK-RR alignment.
